# Economic Analysis of Offering Different Herbage Allowances to Dairy Cows Fed a Partial Mixed Ration

**DOI:** 10.3390/ani11061704

**Published:** 2021-06-07

**Authors:** Christie K. M. Ho, Martin J. Auldist, Marlie M. Wright, Leah C. Marett, Bill Malcolm, William J. Wales

**Affiliations:** 1 Agriculture Victoria, 5 Ring Road, Bundoora VIC 3083, Australia; 2 Agriculture Victoria, 1301 Hazeldean Road, Ellinbank VIC 3821, Australia; martin.auldist@agriculture.vic.gov.au (M.J.A.); marlie.wright@agriculture.vic.gov.au (M.M.W.); leah.marett@agriculture.vic.gov.au (L.C.M.); bill.wales@agriculture.vic.gov.au (W.J.W.); 3 Centre for Agricultural Innovation, The University of Melbourne, Parkville VIC 3010, Australia; 4 Faculty of Veterinary and Agricultural Sciences, The University of Melbourne, Parkville VIC 3010, Australia; b.malcolm@unimelb.edu.au

**Keywords:** marginal response functions, profitability, pasture allowance

## Abstract

**Simple Summary:**

In south-eastern Australia, most dairy cows consume grazed pasture, cereal grain fed in the dairy and hay in the paddock. Previous research has shown that feeding supplements to grazing cows as a well-formulated mixed ration can increase feed intake, milk production and profit. This previous work was conducted under a limited herbage allowance to represent the conditions of drought or a high stocking rate. Two subsequent animal experiments were performed, one in early lactation and the other in late lactation, where the herbage allowance was varied from low to high and used to investigate the economics of partial mixed ration (PMR) feeding. We found that offering a medium allowance (25 and 20 kg DM/cow per day in early and late lactation, respectively) resulted in higher profit (total milk income minus feed costs) than a low herbage allowance (15 and 12 kg DM/cow per day in early and late lactation). No additional profit was obtained by further increasing the herbage allowance from medium to high (40 and 32 kg DM/cow per day in early and late lactation). These findings will assist farmers to manage their PMR systems in a profitable way.

**Abstract:**

The economics of grazing dairy cows offered a range of herbage allowances and fed supplements as a partial mixed ration (PMR) were examined where profit was defined as the margin between total milk income and the cost of pasture plus PMR supplement. The analysis made use of milk production and feed intake data from two dairy cow nutrition experiments, one in early lactation and the other in late lactation. In early lactation and at a PMR intake of 6 kg DM/cow per day, the profit from the cows with access to a medium herbage allowance (25 kg DM/cow per day) was AUD 1.40/cow per day higher than that for cows on a low allowance (15 kg DM/cow per day). At a higher PMR intake of 14 kg DM/cow per day, the profit from the cows on a medium herbage allowance was AUD 0.45/cow per day higher than the cows on a low allowance; there was no additional profit from increasing the herbage allowance from medium to high (40 kg DM/cow per day). In late lactation, the profit from the cows fed a PMR with a medium herbage allowance (20 kg DM/cow per day) was only higher than the cows on a low allowance (12 kg DM/cow per day) when the PMR intake was between 6 and 12 kg DM/cow per day. There was also a difference of AUD +0.50/cow per day between the PMR with medium and high herbage allowance (32 kg DM/cow per day). It was concluded that farmers who feed a PMR to dairy cows should offer at least a medium herbage allowance to optimize profit. While feeding additional PMR increases milk production and profit, further gains would be available by offering a higher herbage allowance. These findings provide an estimate of the net benefits of different herbage allowances when feeding a PMR and will enable farmers to manage their feeding systems more profitably.

## 1. Introduction

Dairy feeding systems in south-eastern Australia are diverse, but on most farms cows graze pasture and are supplemented with cereal grain or pelleted concentrates fed in the dairy [1,2]. Recent research has shown that dairy cows grazing pasture and supplemented with a partial mixed ration (PMR) can increase dry matter (DM) intake and produce more milk than cows fed supplements as cereal grain in the dairy and forage in the paddock [3,4,5,6,7].

When the herbage allowance was restricted, to represent the conditions of drought or a high stocking rate, a PMR diet comprising wheat grain (38% DM basis), maize grain (18%), canola meal (22%) and lucerne hay (22%) fed as a mix on a feed pad has been estimated to contribute AUD 0.97/cow per day more to profit (the margin between total milk income and feed costs) than a conventional diet of wheat grain offered in the dairy and pasture silage in the paddock [8]. The canola meal in the mixed ration appears to motivate cows to consume more pasture and consequently increase milk production [4]. Possible biological mechanisms for this effect include that the high-protein canola acts as a buffer and stabilises rumen pH, that it improves the balance in the supply of amino acids which increases milk production and drives DM intake, or that the removal of cereal grain reduces the build-up of propionate in the rumen and lessens the satiety signals via the hepatic oxidation theory [9,10,11].

To investigate further the impacts of canola meal and increased pasture DM intake, Auldist et al. [12] tested the effects of varying the herbage allowance on the milk production of dairy cows offered 6, 10, 12 or 14 kg DM/cow per day of PMR in early lactation. They found that when cows were offered the same amount of PMR, cows grazing a low herbage allowance of 15 kg DM/cow per day produced less milk and less energy-corrected milk (ECM) than cows on a medium (25 kg DM/cow per day) or high (40 kg DM/cow per day) herbage allowance. There was no difference in the milk fat concentration between the cows grazing the different herbage allowances at any amount of PMR, except at 14 kg DM/cow per day where the milk fat concentration was lower for cows offered the high allowance. Milk fat yield was lower for cows fed 6 kg DM/cow per day of PMR and grazing the low allowance than medium or high allowance, but there were no differences at higher amounts of PMR. Protein concentration and yield were higher for cows on the higher herbage allowances than on the low allowance for all amounts of PMR, with little difference between cows on medium or high allowance.

The experiment by Auldist et al. [12] was followed by a similar experiment in late lactation. The aim was to measure the milk production of cows offered different herbage allowances over a range of PMR intakes. The results of that late lactation study and an economic analysis of PMR feeding where the herbage allowance varied in both early and late lactation are reported here. Although there may be an increase in milk production after implementing a change in feeding regimen, management strategy or adopting new technology, an important consideration for farmers is whether the extra benefits of making the change exceed the extra costs. It was expected that the experiment would show that cows offered an increasing herbage allowance in late lactation would produce more milk with the increase in milk declining as the amount of PMR increases. It was also expected that the economic analysis would show that cows grazing a higher herbage allowance in either early or late lactation would be more profitable than cows on low herbage allowance.

## 2. Materials and Methods

The economic analysis presented here draws on data from two dairy cow nutrition experiments conducted at the Agriculture Victoria research farm at Ellinbank, Victoria, Australia (latitude 38°14′ S, longitude 145°56′ E). The first of these experiments was conducted in spring 2013. This study has been reported in detail by Auldist et al. [12] and is described briefly here. Milk production was measured in Holstein-Friesian cows offered low, medium or high allowances of perennial ryegrass (*Lolium perenne*) pasture (15, 25 or 40 kg DM/cow per day measured to ground level; 2.7%, 4.5%, and 7.2% of animal liveweight) in combination with different amounts of supplement (6, 10, 12 or 14 kg DM/cow per day offered as a PMR; 1.1%, 1.8%, 2.2% and 2.5% of animal liveweight). The 27-day experiment used 144 cows in early lactation (45 ± 17.3 days in milk) and comprised a 14-day adjustment period followed by a 13-day measurement period. Cows had a bodyweight of 558 ± 60 kg immediately before the start of the experiment. The cows were allocated into 24 groups of six, then two groups of six cows received one of the 12 possible combinations of herbage allowance and PMR amount. Each group grazed their allocated area of pasture in separate paddocks. Herbage allowance was measured on every day of the experimental period with the required allowance set by changing the area allocated. The area of pasture allotments was 0.03, 0.05 and 0.08 ha for the low, medium and high allowances, respectively. Cows received their pasture in two fresh allocations per day and were prevented from accessing areas that had been previously grazed. The PMR comprised milled wheat grain (38%, DM basis), crushed maize grain (18%), lucerne hay (22%) and canola meal (22%) and was presented to cows on a feed pad twice daily after the morning and afternoon milking. Group intakes of pasture and supplement were measured daily during the measurement period while milk yield was measured at each milking. Concentrations of fat and protein were measured using an infrared milk analyzer (Model 2000, Bentley Instruments, Chaska, MN, USA) on six days (12 milkings) during the measurement period. 

The second experiment, conducted in autumn 2014, has not been described previously but was identical to the first experiment except that cows were later in lactation (242 ± 20.5 days in milk). Cows had a bodyweight of 589 ± 54 kg immediately before the start of the experiment. The target herbage allowances were 12, 20 and 32 kg DM/cow per day (2.0%, 3.4% and 5.4% of animal liveweight) measured to ground level for low, medium and high allowance, respectively; and the amounts of PMR offered were 6, 8, 10 and 12 kg DM/cow per day (1.0%, 1.4%, 1.7% and 2.0% of liveweight). The appropriate allowance was achieved by altering the area of the allotments. The areas allotted were 0.03, 0.04 and 0.07 ha for low, medium and high allowance, respectively. Cows received pasture as two fresh allocations per day and could not re-graze areas previously grazed. Pre- and post-grazing herbage mass was estimated using a rising plate meter, Ellinbank Plate Meter [13]. Each day of the measurement period, for each group of six cows, 50 readings were taken pre- and post-grazing for each allotment of pasture for low and medium allowance treatments, and 100 readings were taken pre- and post-grazing for each allotment of pasture for high allowance. The pasture meter was calibrated for each new set of paddocks the cows entered by using quadrant cuts to construct calibration equations plotting actual herbage mass to ground level against pasture meter reading. This information was used to calculate average herbage DM intake for each group. The second experiment was 28 days in length, comprising a 14-day adjustment period followed by a 14-day measurement period.

Statistical analyses of both experiments were conducted as described by Auldist et al. [12], where the data were analysed using Genstat 18 software [14]. Milk production data were averaged for each cow within the covariate period and within the measurement period, then averaged within groups of six cows. Intake data for the measurement period were also averaged within groups. The group-averaged milk production data were subjected to ANOVA with the covariate as the corresponding variable from the covariate period. The treatment structure was a two-way factorial, herbage allowance by PMR amount. Intake data were subjected to ANOVA with the same treatment structure, without consideration of a covariate effect. Significant ANOVA for main effects were further examined by l.s.d. tests (α = 5%). There was no blocking structure, corresponding to the completely randomized design, group being the experimental unit. Distributional assumptions of normality and constant variance were checked graphically using plots of residuals against fitted values, normal quantile plots and histograms of residuals. Energy-corrected milk (ECM) was calculated using the formula in Equation (1) [15].

ECM (kg/cow per day) = milk yield kg × (376 × fat% + 209 × protein% + 948)/3138(1)

### Economic Analysis

The approach used in the economic analysis was similar to Ho et al. [8] where it was assumed the infrastructure and equipment needed to mix and feed out a mixed ration were already present on the farm. That is, the additional costs that needed to be accounted for were the costs of the supplement and pasture, and the only change was the amount fed rather than any decision to invest in the capital for a mixed ration system. The economic analysis of a PMR system as an investment decision where capital, machinery and equipment purchases and other changes to the farm system that may be needed to implement a mixed ration feeding system is reported by Henty et al. [16].

Milk production response functions to mixed ration feeding were developed from the early lactation experiment reported in Auldist et al. [12] and the late lactation experiment described above. Trendlines were fitted through the data using Microsoft Excel to establish the marginal milk response relationships with changing PMR intake at different herbage allowances. In this analysis, the profit indicator used was total milk income from milk produced minus the costs of supplement and pasture for different amounts of PMR intake. Total milk income comprised the separate contributions from milk protein and milk fat. Supplement and herbage DM intake data were taken from the early lactation experiment described in Auldist et al. [12] and the results from the late lactation experiment and used to calculate total feed costs at each amount of PMR. The income from milk produced and cost of feed was expressed in Australian dollars (AUD; average exchange rate at the time of analysis: AUD 1 = USD 0.75).

The milk prices and feed costs used to estimate addition to profit were based on historical data from Dairy Australia [17,18] and ABARES [19], with input from a group of industry experts comprising farmers, service providers, scientists and economists (Table 1). A milk price of 6.04 AUD/kg protein + fat (9.00 AUD/kg protein and 3.60 AUD/kg fat) was used, which represents the average of typical factory prices paid in Victoria between 2000 and 2018, after adjusting for inflation [20]. Dairy service levies of 6.99 c/kg protein and 2.87 c/kg fat and volume charges of 2.5 c/kg milk (7.23 c/kg protein + fat energy-corrected milk, 0.38 c/L) were accounted for. Feed prices for wheat grain, maize grain, canola meal and lucerne hay were based on the mean of distributions derived from weekly prices between 2013 and 2018, converted to 2018 dollars. A distribution was fitted around the weekly prices using @Risk, an add-in package to Microsoft Excel [21]. A distribution of prices for maize grain was developed from annual prices between 2013 and 2018 [19] and the mean used in the economic analysis. Pasture is an intermediate input within the farm system, grown to produce livestock or livestock products, such as milk and wool, rather than an output that is directly traded. Pasture can therefore be difficult to value [22]. In the absence of historical data being available to develop a price distribution, the panel of industry experts estimated an average price for pasture to use in the analysis, drawing on market values of pasture and its close substitutes (Table 1).

## 3. Results

### 3.1. Early Lactation

Milk production and intake data from the early lactation experiment were reported by Auldist et al. [12], therefore only the results from the economic analysis of the early lactation experiment are reported below.

#### Economics

**Figure 1:**
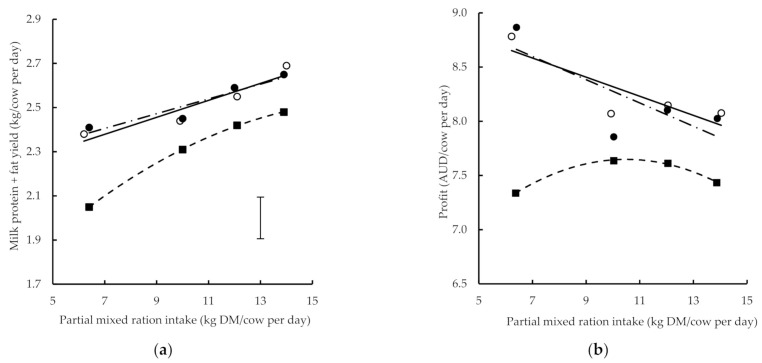
(**a**) Milk protein plus fat production and (**b**) profit (total milk income minus feed cost in AUD) with changing partial mixed ration intake in early lactation where herbage allowance was low (■), medium (◯) or high (●). Lines represent fitted relationships for low (line with short dashes), medium (solid line) and high (dot-dash line) herbage allowance. Vertical bracket represents least significant difference (*p* = 0.05).

The milk response curves for different amounts of PMR for a low, medium and high herbage allowance were developed from the milk protein and milk fat yield data previously given in Auldist et al. [12] (Figure 1). The equations for the trendlines fitted are shown in Table 2. Increasing the herbage allowance offered to cows increased milk protein + fat yield at all levels of PMR. There were no significant interactions between the effects of the allowance and PMR amount [12]. Addition to profit (the margin between total milk income and feed costs) was also higher for the cows that grazed the medium and high herbage allowance than those on the low herbage allowance, at all amounts of PMR intake (Figure 1). However, there was no additional advantage for profit by increasing from medium to high herbage allowance.

The profit from the cows that grazed a medium or high herbage allowance decreased linearly as the amount of PMR offered increased (Figure 1). At 6 kg/cow per day of PMR, profit was AUD 8.81/cow per day compared with AUD 7.80/cow per day at 14 kg DM/cow per day of PMR. The profit from the cows that grazed a low herbage allowance had a quadratic relationship with the changing amount of PMR and the maximum profit for the amounts of PMR offered was AUD 7.62/cow per day. This occurred at 9.7 kg DM/cow per day of PMR offered. Beyond this point, the marginal cost of the extra feed exceeded the marginal revenue from the milk produced.

Compared with the cows grazing the low herbage allowance when 6 kg DM/cow per day of PMR was fed, the profit from the cows grazing a medium or high herbage allowance was AUD 1.40/cow per day higher. At a higher PMR of 14 kg DM/cow per day, the profit from the cows grazing the medium or high herbage allowances was only AUD 0.45/cow per day higher than the cows grazing the low herbage allowance.

### 3.2. Late Lactation

#### 3.2.1. Feed Intake

Pasture DM intake increased as herbage allowance increased, but the amount of PMR offered did not have a significant effect on pasture DM intake (Table 3). Pasture utilisation decreased with an increasing herbage allowance and with an increasing amount of PMR offered. At low, medium or high herbage allowance, supplement DM intake increased with an increasing amount of PMR offered. The total DM intake increased with increasing herbage allowance and as more PMR was offered.

#### 3.2.2. Milk Yield and Composition

The milk yield and ECM yield of cows grazing a low, medium or high herbage allowance increased as the amount of PMR offered increased (Table 4). When 6, 8 or 10 kg DM PMR/cow per day was offered, the difference in milk yield or ECM yield between cows grazing a low herbage allowance and a medium or high allowance ranged between 1.8 and 5.3 kg. When 12 kg DM/cow per day of PMR was fed, the difference in milk yield or ECM yield was smaller amongst cows grazing low and medium or high herbage allowance. The difference in milk yield and ECM yield of cows on medium or high allowance was less than 1.8 kg at all amounts of PMR offered.

Milk fat concentration was highest for cows grazing the low herbage allowance at all amounts of PMR, but cows on all herbage allowances showed a decreasing trend in milk fat concentration as the amount of supplement offered increased. The milk fat yield for cows on medium or high herbage allowance was higher than that for cows grazing the low allowance, except when 12 kg DM PMR/cow per day was offered, where milk fat yield was the same for all herbage allowances.

There was no clear trend in the milk protein concentration when the amount of PMR offered changed or when herbage allowance varied. However, in a similar way to milk fat yield, milk protein yield for cows grazing the medium and high herbage allowance was clearly higher than for cows grazing the low allowance. There was no difference in the milk protein yield of cows grazing the medium or high allowance at any amount of PMR offered.

#### 3.2.3. Economics

**Figure 2:**
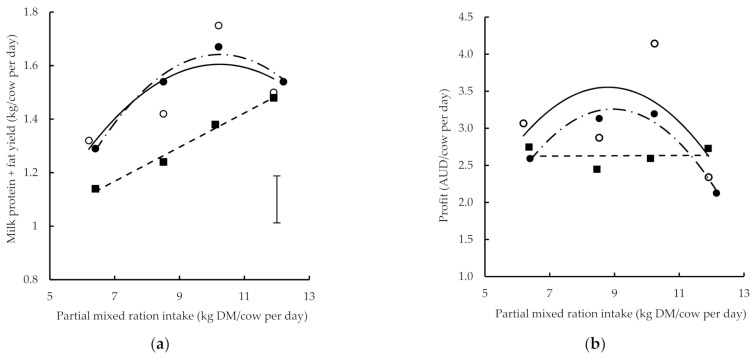
(**a**) Milk protein plus fat production and (**b**) profit (total milk income minus feed cost in AUD) with changing partial mixed ration intake in late lactation where herbage allowance was low (■), medium (◯) or high (●). Lines represent fitted relationships for low (line with short dashes), medium (solid line) and high (dot-dash line) herbage allowance. Vertical bracket represents least significant difference (*p* = 0.05).

The addition to profit of cows in late lactation, measured as the margin of total milk income minus feed costs, increased with the increasing herbage allowance. The profit of cows that grazed medium and high herbage allowances had a negative quadratic relationship with the amount of PMR offered (Figure 2, Table 2). The maximum profit for cows grazing the medium and high herbage allowances was AUD 3.48/cow per day and AUD 3.18/cow per day, respectively. This occurred where the amounts of PMR were 8.6 kg DM/cow and 8.8 kg DM/cow per day. The profit of cows grazing the low herbage allowance did not change with an increasing amount of PMR (Figure 2).

Increasing herbage allowance increased profit compared with cows grazing the low herbage allowance, but only where the PMR offered was between 6 and 12 kg DM/cow per day. The maximum difference in profit between medium and low herbage allowance was AUD 0.98/cow per day when 8.6 kg DM/cow per day of PMR was fed. At the high herbage allowance, the largest difference was AUD 0.68/cow per day when the amount of PMR was 8.8 kg DM/cow per day. There was a difference of approximately AUD 0.50/cow per day in profit between cows grazing medium and high herbage allowance over the range of PMR amounts tested (Figure 2).

## 4. Discussion

The experiment reported here has measured the impact of varying the herbage allowance on the milk production of cows fed different amounts of PMR in late lactation. As hypothesized, the yields of milk, ECM, milk fat and milk protein were higher for cows offered a medium or high herbage allowance than cows grazing the low herbage allowance, at all amounts of PMR offered. Milk fat concentration was also higher for cows on medium or high herbage allowance except when 6 kg DM/cow per day of PMR was fed. There were no differences in the milk protein concentration of cows on the different herbage allowances at any amount of PMR. There was also little difference in milk production between cows grazing the medium or high herbage allowance. This finding was similar to that of cows in early lactation [12]. Except for cows grazing the low herbage allowance in late lactation, marginal milk responses declined with increasing amounts of PMR and the decline was greater when the herbage allowance was medium or high. This effect of diminishing returns with increasing DM intake has been reported in previous studies [23,24].

The contribution to profit, considered in this study as the margin between total milk income and feed costs, was higher for cows that grazed medium or high herbage allowance than low herbage allowance in both early and late lactation, supporting the second hypothesis, but the magnitude differed. In early lactation, the difference in profit between cows that grazed the low herbage allowance and those that grazed the medium or high herbage allowance was greater at lower amounts of PMR than at higher amounts of PMR. The higher herbage allowances also increased profit in late lactation, but only where the amount of PMR fed was between 6 and 12 kg DM/cow per day. Outside this range, the trendlines fitted indicated that profit would be lower for the higher herbage allowances. There was no difference in the milk protein + fat yield of cows grazing the medium and high herbage allowances in either early or late lactation, and consequently, there was no additional profit. While cows consumed more pasture when offered the high allowance, this did not increase milk production or profit in either early or late lactation. However, the other potential benefits of offering a high herbage allowance in addition to milk production, such as for body condition and reproduction, were not valued in the analysis.

The response of profit to changing PMR intake was a negative quadratic relationship when cows grazed a low herbage allowance in early lactation and a medium or high allowance in late lactation. In these situations, the profit maximizing amount of PMR could be calculated, i.e., the level of PMR where the extra costs of the feed equaled the extra revenue from the milk produced [25,26]. For the remaining cases, when cows were offered a medium and high allowance in early lactation and a low herbage allowance in late lactation, the range in the amount of supplement tested in the experiments did not include the point of diminishing returns. As a result, profit had a linear relationship with PMR intake and the profit maximizing amount of PMR to feed could not be estimated.

The implications of the results from the experiment and economic analysis described here are that farmers who feed a PMR to their dairy cows should offer at least a medium herbage allowance to optimize profit. At the low allowance, the feeding of additional PMR increases milk production and profit, but further gains would be available by offering a higher herbage allowance. For example, in early lactation when the amount of PMR fed increased from 6 to 10 kg DM/cow per day for cows grazing a low herbage allowance, profit increased by AUD 0.30/cow per day (7.35 to 7.65 AUD/cow per day). However, increasing from a low to medium herbage allowance at 6 kg DM/cow per day of PMR, increased profit by AUD 1.45/cow per day to AUD 8.80/cow per day. Where the amount of PMR fed was between 6 and 12 kg, profit was also higher in late lactation when the herbage allowance increased from low to medium or high allowance, compared with when the amount of PMR was increased and cows grazed a low allowance. In the temperate dairy regions of Australia where perennial ryegrass is the dominant pasture species, dairy farm managers may need to consider making use of autumn active crops or alternative forages such as chicory to fill summer−autumn feed gaps when perennial pasture is limited [27,28,29].

The experiments used for the economic analysis were conducted under conditions where a consistent herbage allowance was offered and the ability to do this could be a critical factor for achieving the full economic benefit. If the herbage allowance varies from low to high, the benefits estimated here may be reduced. The impact of variable herbage allocation on milk production and profit has been previously demonstrated, with a more consistent herbage allocation shown to increase milk yield by 9% for cows grazing ryegrass pasture [30]. In a modelling study that investigated different levels of knowledge about pasture mass, Beukes et al. [31] found that annual farm operating profit could be increased by 11–15% if pasture mass could be estimated with an error of 15% of less. The main factor identified as leading to higher farm profit was more accurate herbage allocation which reduced the likelihood of under or over-grazing. Over 70% of Australian dairy farmers currently make decisions about grazing pastures based on past experience or intuition [32], but as the availability, timeliness and accuracy of pasture measurement tools and devices improve, the uptake of these technologies could assist with more evenly allocating pasture to grazing dairy cows, particularly for farms with larger herd sizes [31,33,34,35].

## 5. Conclusions

Cows fed a PMR with access to a medium herbage allowance (25 kg DM/cow per day) in early lactation were found to have higher profit than cows on a low herbage allowance (15 kg DM/cow per day). No additional profit was obtained by increasing the herbage allowance from medium to high (40 kg DM/cow per day). In late lactation, cows fed a PMR and offered a medium herbage allowance (20 kg DM/cow per day) only contributed more to profit than cows fed PMR with low herbage allowance (12 kg DM/cow per day) when their supplement intake was between 6 and 12 kg DM/cow per day. In addition, there was a difference of AUD +0.50/cow per day between cows on a medium and high herbage allowance (32 kg DM/cow per day). It is concluded that farmers who feed a PMR to dairy cows in early or late lactation should offer a medium herbage allowance to optimize profit. These results provide farmers operating PMR systems with valuable information about how to profitably manage mixed ration feeding with grazed pasture.

## Figures and Tables

**Table 1:** Milk prices and feed prices used in the economic analysis (in AUD; average exchange rate at the time of analysis: AUD 1 = USD 0.75).

Item	Prices Received or Paid
Milk protein and fat	9.00 AUD/kg protein, 3.60 AUD/kg fat
Grain	343 AUD/t DM wheat, 439 AUD/t DM maize
Canola meal	486 AUD/t DM
Lucerne hay	391 AUD/t DM
Pasture	150 AUD/t DM

**Table 2:** Equations describing the relationships between partial mixed ration (PMR) dry matter intake (DMI; kg DM/cow per day) and milk protein plus fat yield (PY + FY) and profit (total milk income minus feed costs) for cows offered low, medium and high herbage allowance ^1^.

Parameter	Herbage Allowance	Equation	R^2^
Early lactation			
PY + FY (kg/cow per day)	Low	PY + FY = −0.0039DMI^2^ + 0.137DMI + 1.333	0.9999
	Medium	PY + FY = 0.0385DMI + 2.1093	0.8934
	High	PY + FY = 0.0334DMI + 2.1714	0.8913
Profit (AUD/cow per day)	Low	Profit = −0.019DMI^2^ + 0.3684DMI + 5.8380	0.9970
	Medium	Profit = −0.1417DMI + 9.7098	0.7051
	High	Profit = −0.1216DMI + 9.5436	0.8319
Late lactation			
PY + FY (kg/cow per day)	Low	PY + FY = 0.0637DMI + 0.7220	0.9871
	Medium	PY + FY = −0.0195DMI^2^ + 0.3983DMI − 0.4333	0.5988
	High	PY + FY = −0.0243DMI^2^ + 0.4987DMI − 0.9127	0.9775
Profit (AUD/cow per day)	Low	Profit = −0.0295DMI + 2.7681	0.2054
	Medium	Profit = −0.0998DMI^2^ + 1.7255DMI − 3.9796	0.3342
	High	Profit = −0.1104DMI^2^ + 1.9413DMI − 5.3549	0.9688

^1^ Equations derived from trendlines fitted to the mean measured values at four amounts of PMR per feeding strategy.

**Table 3:** Dry matter intake (kg DM/cow per day) from pasture and supplement for cows in late lactation offered a low, medium and high herbage allowance (kg DM/cow per day) and offered a partial mixed ration (PMR) at nominal amounts of 6, 10, 12 or 14 kg DM/cow per day. Data are means from the 14-day measurement period.

Herbage Allowance	PMR Offered	Herbage Allowance	PMR Intake	Pasture Intake
Low	6	11.9	6.4	8.7
	8	11.9	8.5	8.7
	10	12.0	10.1	8.8
	12	10.9	11.9	7.6
Medium	6	19.1	6.2	12.7
	8	20.0	8.5	13.2
	10	20.0	10.2	12.7
	12	19.4	11.9	12.3
High	6	30.3	6.4	15.8
	8	30.4	8.5	16.5
	10	31.7	10.2	16.5
	12	31.1	12.2	15.5
*p* (allowance)		<0.001	0.029	<0.001
*p* (PMR) ^1^		0.661	<0.001	0.706
S.E.D.		1.215	0.084	1.596
L.S.D.		2.648	0.183	3.478

^1^ There were no significant interactions between the effects of allowance and PMR amount.

**Table 4:** Mean yields (kg/cow per day) of milk (MY) and energy-corrected milk (ECM), and concentrations (%) and yields (kg/cow per day) of milk fat and protein of cows in late lactation offered low, medium and high herbage allowance and a partial mixed ration (PMR) offered at different amounts (kg DM/cow per day). Data are means from the 14-day measurement period.

Herbage Allowance	PMR Offered	MY	ECM	Fat	Fat Yield	Protein	Protein Yield
Low	6	14.5	15.3	4.31	0.62	3.65	0.53
	8	15.4	16.6	4.46	0.68	3.65	0.56
	10	17.6	18.5	4.26	0.75	3.54	0.62
	12	19.0	19.7	4.21	0.79	3.62	0.68
Medium	6	17.0	17.5	4.13	0.69	3.58	0.60
	8	18.6	19.1	4.08	0.75	3.60	0.67
	10	22.9	23.5	4.14	0.93	3.62	0.82
	12	20.0	20.3	4.09	0.80	3.63	0.71
High	6	16.7	17.4	4.22	0.70	3.67	0.61
	8	20.4	20.8	3.97	0.81	3.60	0.73
	10	22.2	22.4	3.98	0.87	3.63	0.80
	12	21.0	21.1	3.93	0.81	3.65	0.75
*p* (allowance)		<0.001	<0.001	0.005	0.018	0.627	<0.001
*p* (PMR) ^1^		<0.001	<0.001	0.280	<0.001	0.745	<0.001
S.E.D.		1.419	1.431	0.144	0.060	0.052	0.052
L.S.D.		3.122	3.150	0.317	0.133	0.114	0.115

^1^ There were no significant interactions between the effects of allowance and PMR amount.
